# Memory Training Program Decreases the Circulating Level of Cortisol and Pro-inflammatory Cytokines in Healthy Older Adults

**DOI:** 10.3389/fnmol.2017.00233

**Published:** 2017-07-24

**Authors:** Mirko Pesce, Raffaella Tatangelo, Irene La Fratta, Alessia Rizzuto, Giovanna Campagna, Cinzia Turli, Alessio Ferrone, Sara Franceschelli, Lorenza Speranza, Maria C. Verrocchio, Maria A. De Lutiis, Mario Felaco, Alfredo Grilli

**Affiliations:** School of Medicine and Health Science, University G. D’Annunzio Chieti, Italy

**Keywords:** Memory Training, cortisol, inflammaging, WIP-1, pro-inflammatory cytokines

## Abstract

Aging cognitive decline has been associated to impairment of the Hypothalamus Pituitary Adrenals (HPA) axis activity and a higher level of the systemic inflammation. However, little is known about the molecules driving this process at peripheral level. In addition, the cognitive function is to some extent modifiable with Memory Training (MT) programs, even among older adults and beyond. The study aims to evaluate whether MT could contribute to ameliorate cognitive performance and modulate the HPA axis activity as well the low level inflammation in the aging phenotype. Whether the phosphatase WIP-1, a negative regulator for inflammation, is involved in this process was also investigated. We recruited 31 young adults (19–28, years of age) and 62 older adults aged over 60. Thirty-two older adults were submitted to 6-months of MT program (EG), and 28 older adults were no treated and used as Control Group (CG). Global cognitive functioning (MMSE score), verbal and visual memory, and attention were assessed at baseline (T0) and after 6-months (T1). At the same time, plasmatic level of Cortisol (C), IL-1β, IL-18, IL-6, and the expression of WIP-1 mRNA and protein in *ex vivo* Peripheral Blood Mononuclear Cells were analyzed in young adults at T0, as well in older adults at T0 and T1. Together, the results suggest that MT improves the global cognitive functionality, verbal and visual memory, as well as the level of attention. At the same time we observed a decrease of the plasmatic level of C, of the cytokines, and an increase of the expression of mRNA and protein of WIP-1. The analysis of correlations highlighted that the level of the mRNA of WIP-1 was positively associated to the MMSE score, and negatively to the C and cytokine levels. In conclusion, we purpose the MT as tool that could help support successful aging through the improving of memory, attention and global cognitive function performance. Furthermore, this approach could participate to maintain lower the peripheral levels of the C and pro-inflammatory cytokines. The WIP-1 as a potential new target of the pathophysiology of aging is theorized.

## Introduction

During adulthood, the cognitive performance and immunity system begins to decline concurrently prior to the third decade of life suggesting that the mechanisms underlying these processes are inter-related (Rosano et al., 2012). There is comparable evidence indicating that increases in the level of peripheral markers of inflammation are associated with age-related declines in the health of the brain (Franceschi, 2007). Such evidence is parallel to longstanding findings from neurocognitive studies in populations of older adults living in a community, that indicate in a consistent manner an association between higher inflammatory levels and lower cognitive levels and a higher risk of cognitive impairment over time (Gorelick et al., 2014).

The systemic inflammation related to aging involves multiple organs and represents a common mechanism leading to multiple chronic diseases such as dementia, diabetes, atherosclerosis, and arthritis, which has a negative impact on the health span of the elderly (Maggio et al., 2005; Franceschi, 2007; Green et al., 2011). These findings supported the ‘inflammaging theory’ that hypothesizes a global reduction in the capacity to cope with a variety of stressors and purposes a concomitant progressive cytokines dysregulation as the major characteristics of the aging process (Franceschi and Campisi, 2014).

The modification of the Hypothalamus Pituitary Adrenal (HPA) axis activity represents another hallmark of the aging phenotype (Ferrari and Magri, 2008). Several findings suggest that a higher basal endogenous cortisol (C) concentration, a downstream effect of the increase in basal tonus of the HPA axis activation, is age-related (Deuschle et al., 1997; Giordano et al., 2005). The enhanced HPA axis activity could be explained by a decrease of the hippocampal inhibitory function that leads to an increase of the release of C (De Kloet et al., 1998). Consistently, the aging related higher plasma levels of C have predicted a smaller hippocampal volume, faster hippocampal atrophy (Lupien et al., 1998; O’Hara et al., 2007), that are associated to memory decline, and to general poor cognitive performance (Lupien et al., 2007; Lee et al., 2008; Tatomir et al., 2014; Geerlings et al., 2015; Vogel et al., 2016).

The aging systemic higher exposure to C could be associated with the accelerated features of low-grade inflammation that characterizes older adults. The HPA axis activity exerts a pivotal role for the homeostasis of the immune system. The circulating level of C and those of several cytokines have been concurrently associated with a decreased hippocampal volume and cognitive impairment (Bauer, 2008; Sudheimer et al., 2014; Metti et al., 2015; Chesnokova et al., 2016; Jin et al., 2016). The Glucocorticoids (GCs) and pro-inflammatory cytokines are not independent of each other and they interact on multiple levels. In particular, the GCs that were classically viewed as anti-inflammatory could have a dual role, inducing pro-inflammatory effects. Their administration results in an increased systemic trafficking of lymphocytes and monocytes (Bowers et al., 2008), and the exertion of pro-inflammatory actions through synergistic interactions with inflammatory cytokines, such as IL-1β and IL-6 (Langlais et al., 2008; Ding et al., 2010; Busillo et al., 2011).

The mechanisms underlying low-grade systemic inflammation in several organs in the absence of overt infection that was observed during aging are still speculative, and little is known about the “master” molecule involved in this process. The Wild type p53-Induced Phosphatase-1 (WIP-1) is a phosphatase whose expression is induced in response to many types of cellular stress, as γ or UV radiation in a p53-dependent manner (Fiscella et al., 1997). This protein has been associated with cellular senescence (Salminen and Kaarniranta, 2011), and its expression has been showed to decrease during age (Wong et al., 2009). More recently, WIP-1 has been recognized as an intrinsic negative regulator of inflammatory response in the CNS (Tan et al., 2013), and also at the peripheral level where it acts as a negative regulator for neutrophil migration and pro-inflammatory cytokines production through various pathways, including the feedback regulation of the NF-κB signaling (Sun et al., 2014). It could be hypothesized that WIP-1 could be associated with sterile inflammation that underlines the aging process.

The above mentioned age-related modulations and variations are dynamic and not irreversible processes. The ways that allow modulation of brain plasticity in animal and human using cognitive or physical training have resulted to be of great interest (Curlik and Shors, 2013). Several studies on rodents have shown that Environmental Enrichment (EE) – modification of the environment to enhance the animal physical and psychological well-being – (Baumans, 2005), has been shown to slow down neuronal aging (Kempermann et al., 2002), improve cognition and memory (Jankowsky et al., 2005; Hannan, 2014). Some EE stimuli are able to affect C (Simpson and Kelly, 2011), cytokines and various immune components at a peripheral level, suggesting that this could be a potential mechanism of action in how it could modulate the brain function (Singhal et al., 2014). To this end, within cognitive training procedures, Memory Training (MT) based research generally reveals an improvement in memory performance in older adults through the utilization of mnemonic strategies. However, even cognitive training was successfully included within EE stimuli. To our knowledge, neither study has been carried out to determine whether it reduces the endogenous C levels and the behavioral sequence of chronic inflammatory conditions that characterize the aging phenotype.

Considering the above mentioned in aggregate, we hypothesize that the MT could be considered as a treatment that similar to other stimuli of the EE, could enhance the cognitive and physical well-being of older adults. It is for this reason that this study wants to investigate whether a MT program acts on the plasmatic level of C, the pro-inflammatory cytokines IL-6, IL-1β, and L-18, and the expression of the WIP-1 in *ex vivo* Peripheral Blood Mononuclear Cells (PBMCs) of apparently healthy elderly (older adults, 65–74 years; De Beni, 2009), relating to their degree of cognitive abilities. We chose these cytokines because all of them had been previously associated with specific features of aging phenotype (Franceschi, 2007), and also their higher levels had been successfully suggested as a marker of poorer cognitive performance in healthy subjects for the IL-6 and the IL-1β (Yirmiya and Goshen, 2011), and in a clinical population for the IL-18 (Bossù et al., 2008; Zhang et al., 2013).

Our results provide important evidence that MT could contribute to the decreasing of the C level, restore the WIP-1 expression and reduce the value of the pro-inflammatory cytokines at a peripheral level, participating in “holding off” the systemic inflammatory condition that characterizes healthy older adults. We first involved WIP-1 deficiency in the pathophysiology of aging.

## Materials and Methods

### Participants

Participants of the study were 62 apparent healthy older adult volunteers (29 female, age range 65–74 years of age), recruited with the use of posters, pamphlets and word of mouth at recreational clubs. Thirty-one apparent healthy young adults (16 female, age 19–28 years of age), were also recruited by word of mouth and with pamphlets at University G. d’Annunzio, CH-PE, Italy. The exclusion criteria regarding all participants were summarized in **Table 1**.

**Table 1:** Exclusion criteria of all participants.

Exclusion criteria
• Body Mass Index (<20 and >33 kg/m^2^)
• Erythrocyte Sedimentation Rate (<20 mm/hr for males; <30 mm/hr for females)
• C Reactive Protein (<5 mg/L)
• Hematological and Biochemical Analyses Values within Normal Range
• Unusual Dietary Habits (e.g., vegetarians)
• Recent infections, allergies, present or past autoimmune disorders
• Diagnosis of or treatment for pathological diseases (cancer, diabetes, insufficient renal and hepatic performance, mal-absorption and chronic inflammatory pathologies)
• Current medication (including herbal remedies or vitamins)
• Smoking
• Alcohol consumption (1 week the sample collection, >30 g/d for men and >20 g/d for woman)

Briefly, subjects with the BMI < 20 and >33 k/m^2^ were excluded (Travison et al., 2007; Mangnus et al., 2016). Subjects with unusual dietary habits (e.g., vegetarians) were also excluded (Sutliffe et al., 2015). Hematological and biochemical tests needed to be within the normal range. Negative serologies for the HIV and Hepatitis C viruses were verified. To assess the inflammatory status, all recruited subjects underwent the same laboratory blood tests: Erythrocyte Sedimentation Rate (ESR) and C-Reactive Protein (CRP) were measured as non-specific markers for inflammation and were utilized as exclusion criteria. The upper limit of the ESR is 20 mm/hr, for males, and 30 mm/hr for females (Sox and Liang, 1986; Caswell, 1993). To avoid the inclusion of subjects in an acute phase of inflammation (e.g., infection, trauma and tissue necrosis, malignancies, and autoimmune disorders), the subjects with CRP levels > 5 mg/L were also excluded (Gabay and Kashner, 1999; Ablij and Meinders, 2002; Biasucci, 2004).

Habitual smokers were excluded because this factor has already been significantly marked as a modulator of C level and strong pro-inflammatory (Mendelson et al., 2008; Messner and Bernhard, 2014). The participants were invited not to consume alcohol starting 1 week before the sample collection, in order to avoid any effects on C and the systemic inflammation levels (Sher et al., 2007; Morris et al., 2015).

Subjects who had current infections, allergies, or a present and past history of autoimmune disorders, and those on current medication (including herbal remedies or vitamins) such as anti-inflammatory, antiviral agents or immunosuppressive medication that could directly or indirectly affect the systemic inflammatory state were excluded.

Older adults reaching the “exclusion criteria” adapted from the SENIEUR protocol for demographic suitability was excluded from the study (Ligthart et al., 1984). The exclusion criteria for older adults included factors thought to influence the relationship between the cognitive function and chronic inflammation such as the presence of dementia, and depression. Volunteers were invited to a preliminary screening session based on a full medical history and examination including, anthropometric measurements, the assessment of dietary habits, tobacco and alcohol consumption, and screening for cognitive impairment using the Mini-Mental State Examination (MMSE, Folstein et al., 1975), and the Geriatric Depression Scale (GDS), that provides a screening measure of depressive symptoms in adults over 55 years (Yesavage et al., 1982). All participants had their cognitive functions assessed using a neurocognitive battery of tests as described to follow. The neurocognitive battery was designed in order to evaluate the memory and attention which are frequently affected in older persons. The subjects with depression (score ≥ 11 on the GDS) and subjects with dementia (score ≤ 24 on the MMSE) were excluded.

A total of 94 consecutive older adults (39 female) were invited to participate in the study. Ten subjects (4 female) were excluded as they were smokers; 3 male subjects were excluded because they had elevated ESR values, one female subject for an elevated CRP value. Eight male and 10 female were excluded as they were on medication. In the end, 62 consecutive subjects (of which 66% subjects contacted) were included in the study. After having signed the consent, 34 subjects were randomly assigned to an Experimental Group (EG) (16 female) and 28 to a Control Group (CG) (13 female) before the cognitive screening assessment.

A total of 43 consecutive young adults (20 female) were invited to participate in the study. Eight subjects (2 female) were excluded as they were smokers, four (2 female) were excluded as they were on medication. In the end, 31 consecutive subjects (of which 74% of subjects contacted) were included in the study.

The study procedures were described in detail to all participants, a minimum reflection time of 24 h was given before written informed consent was obtained. Participants not having the capacity to consent to research participation and non-Italian-speaking participants (because the inclusion of these participants would have meant not to be able to use standardized assessment techniques) were also excluded from the study.

The study was conducted according to the principles expressed in the Declaration of Helsinki and subsequent revisions and was approved by the Ethics Committee of G. d’Annunzio University, Chieti, Italy.

### Characteristic of the Sample

Demographic data of the subjects studied are listed in **Table 2**, together with BMI value and plasmatic level of CRP.

**Table 2:** Characteristics of older adults at baseline.

Characteristics	Control Group (*n* = 28) (mean ± SD)	Experimental Group (n = 34) (mean ± SD)	*p*-value
Age (years)	69.0 ± 8.0	70.6 ± 7.0	0.428^a^
Gender (F)	28 (13)	34 (16)	0.730^b^
Education (years)	9.4 ± 1.7	9.6 ± 1.8	0.681^a^
BMI (Kg/m^2^)	26.8 ± 2.7	27.0 ± 3.0	0.824^a^
Plasma CRP (mg/l)	1.7 ± 0.6	1.6 ± 0.6	0.530^a^

*^a^Unpaired Student *t*-test; ^b^Chi-squared test; BMI, Body Mass Index; CRP, C-Reactive Protein.*

The CG included 28 subjects (13 women; age, mean ± SD, 69.0 ± 8.0 years). The EG included 34 subjects (16 women; age, mean ± SD, 70.6 ± 7.0 years).

The two groups were not significantly different at the baseline (T0) in terms of age (*p* = 0.428), male/female ratio (*p* = 0.730), BMI (*p* = 0.841), education (*p* = 0.681), and plasma CRP (*p* = 0.530). They did not significantly differ for any neuropsychological scores obtained at the beginning in which all scores were within the normal range. At this period all participants were considered healthy and defined as not suffering from any recent episode that could condition the basal inflammatory level and cognitive performance (i.e., infection and malignancy).

### Neuropsychological Test Battery

Cognitive functioning in CG and EG was measured using a comprehensive neuropsychological test battery at baseline (T0) and after 6 months (T1), 1 day before blood sample collection. For all subjects and controls, the same test administration order was used. The battery was comprised of tests with strong psychometric properties and assessed the following cognitive domains:

Global Assessment of Cognitive Function. MMSE (Folstein et al., 1975) is the use a psychometric test used to quantify the global cognitive functioning and the cognitive change in population-based longitudinal studies. MMSE consists of a series of questions with the aim of quantifying the global cognitive functioning on a 0–30 scale.

The following instruments were administered (in this fixed order), by a psychologist for the overall assessment of each participant, of which had a duration of approximately 90 min, with breaks provided, as requested by participants:

(1) Global Assessment of Cognitive Function. MMSE (Folstein et al., 1975) is the use a psychometric test used to quantify the global cognitive functioning and the cognitive change in population-based longitudinal studies. MMSE consists of a series of questions with the aim of quantifying the global cognitive functioning on a 0–30 scale.
(2) The GDS was created specifically for screening depressive symptoms among older populations and has been widely utilized in both clinical and research settings. The GDS-30 comprises 30 questions regarding high and low mood, lack of energy, anxiety, and social withdrawal. Assesses depressive symptoms by means of 30 questions to be answered with ‘yes’ or ‘no’. Each question is weighted with 1 point for a positive answer. The sum of these points defines the final score, ranging from 0 to 30 points. The GDS-30 asks how the person feels currently and how the person has felt during the previous week (Yesavage et al., 1982).
(3) The Rey-Osterrieth Complex Figure Test was used to assess visuo-spatial constructional functions, visuographic memory, and some aspects of planning (Reedy et al., 2013). The subject was asked to make a copy of a stimulus drawing comprised of a complex design, which was then scored on 18 identifiable details with a maximum score of 36 points possible and a higher score indicating better performance. The subject then drew the figure from memory 5 min later, and again 30 min later.
(4) Memory. The ability to retain and recall verbal information was measured using the Rey Auditory Verbal Learning Test (Lezak, 1995). Participants read a 15-item word list five times and were asked to repeat as many words as they could remember for each time. After 50 times, participants were presented an interference list. Participants were then asked to recall words from the original list. Participants were also asked to recall words from the original list 20 min later.
(5) Attentive Matrices Test (Attentive Test) is a valid instrument to measure selective and sustained attention (Spinnler and Tognoni, 1987). It consists of 3 numeric matrices (10 columns of 13 numbers from 0 to 9). The participants are required to check specific target numbers in 45 s for each matrix. There are 1 target in the first matrix, 2 in the second one, and 3 in the third one. The score is assigned giving 1 point for each target correctly found in the 3 matrices (max 60 for all the 3 matrices). The time to complete the 3 matrices is considered as scores too.
(6) Trail Making Test (TMT) provides information on visual search, speed of processing, mental flexibility, selective visual attention and ability to shifting (Reitan, 1958). It consists of two parts (A and B), both consisting of 25 circles distributed over a sheet of paper. In Part A, the circles are numbered 1–25, and the subject should draw lines to connect the numbers in ascending order. In Part B, the circles include both numbers (1–13) and letters (A–L) and the subject should draw lines to connect alternatively numbers and letters in ascending order (i.e., 1-A-2-B-3-C). The subject should be instructed to connect the circles as quickly as possible, without lifting the pen or pencil from the paper. Errors should be pointed out immediately and subjects should be allowed to correct it. Time required to complete each of the two parts is recorded, considered as scores of the test.

### Memory Training

The participants of EG were submitted to MT from T0 to T1. The MT is based on the concept that repeated practice within a specific domain results in gains in both cognitive and behavioral efficiency of the targeted domain, as well as subsequently transferring improvements to untrained domains.

**FIGURE 1:**
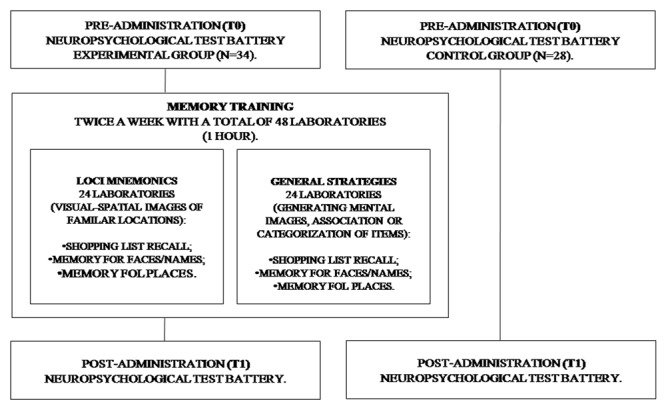
Flow chart of the Memory Training Program.

The intervention was conducted in several laboratories. All sessions lasted 1 h and were held twice a week, with a total of 48 laboratories (**Figure 1**).

During MT, two different mnemonics were used. To assess the EG visual mnemonic (Loci) and general strategies (Strategic) were used (Cavallini et al., 2003). The Loci mnemonics is based on participants that generate sequences of visuo-spatial images of familiar locations, and further produce an image that is more complex, with the initial scene and the name/object to remember. The second training was characterized by the use of disparate strategies in different situations and each of them required a special strategy (generating mental images, association or categorization of items).

Older adults are particularly sensitive to the setting and emotional conditions. For this reason, we used ecological tasks to reproduce daily activities (Baltes and Baltes, 1990).

Story recall. All participants had 5 min to study a short story composed of 22 units. They then had to write what they remembered. Performance was assessed according to the correct number of units they could remember.

Shopping list recall. A shopping list was read to all the participants and they had 5 min to remember and subsequently put it in writing. Performance was evaluated according to the correct number of products which were remembered.

Memory for faces/names. Twelve photographs, each coupled with a name, on a computer screen was presented to all participants for 30 s. The same faces, were then shown without names and participants had to remember the names. Performance was assessed according to the correct face/names associations made.

Memory for places. A map of an Italian city with the name and the position of 10 monuments was presented to all participants for 5 min. They then had to remember and to write the name and the position of the monuments on a blank map. Performance was evaluated according to the correct names and positions which were remembered (Cavallini et al., 2003).

### Cell Sources and Cultures

Blood samples were collected at T0 and T1. Veni-puncture was performed in the morning between 08.00 and 10.00 am. in order to avoid the effect of diurnal variation, and peripheral blood samples were collected in 4 ml endotoxin-free Heparin tubes (Vacutainer, Becton Dickinson, NJ, United States). Tubes were kept at room temperature and transported to the laboratory for processing within 1 h of collection. The plasma was obtained by blood centrifugation as described previously and was kept frozen at -20°C (Pesce et al., 2014).

The PBMCs were isolated as described previously and used freshly for the evaluation of the WIP-1 mRNA and protein expressions and for the study of NF-kB signaling (Speranza et al., 2013). Briefly, the PBMCs were isolated by density-gradient centrifugation through Ficoll-Hypaque (Pharmacia), suspended (10^6^/ml) in RPMI 1640 medium (Sigma-Aldrich, St Louis, MO, United States), containing L-glutamine 1% and antibiotics (penicillin 100 U/ml-streptomycin 100 ug/ml) with 10% heat-inactivated fetal calf serum (Sigma, CA, United States), seeded in polypropylene tubes (Falcon, BD, Lincoln Park, NJ, United States) and incubated at 37°C in a 95% humidified 5% CO_2_ cell culture incubator. Cell viability in each culture was assessed by Trypan blue die exclusion. All solutions were prepared using pyrogen-free water and sterile polypropylene plastic-ware and were free of detectable LPS (<0.1 EU/ml), as determined by the Limulus amoebocyte lysate assay (sensitivity limit 12 pg/ml; Associates of Cape Cod, Falmouth, MA, United States). All reagents used were tested before use for Mycoplasma contamination (minimum detection level 0.1 μg/ml) (Whittaker Bioproducts, Walkersville, MD, United States) and found negative. The same batches of serum and medium were used in all experiments. After 24 h incubation, samples were centrifuged at 400 × *g* for 10 min at room temperature, supernatants were collected and stored at -80°C pending assay (Pesce et al., 2013). The PBMCs yield per mL of blood was approximately 1 × 10^6^ cells.

### RNA Extraction and the Quantitative Real-Time Polymerase Chain Reaction (qPCR) System

Total RNA was extracted from PBMCs of all subjects using the TRIzol reagent (Invitrogen, Life Technologies, Paisley, United Kingdom) according to the manufacturer’s protocol. The RNA concentration and purity were determined by measuring absorbencies at 260 and 280 nm; a 260:280 ratio of 1.9 being considered acceptable for analysis. The RNA concentration was estimated by measuring the absorbance at 260 nm using a Bio-Photometer (Eppendorf AG, Hamburg, Germany), and RNA samples were kept frozen at –80°C until use. Purified RNA was electrophoresed on a 1% agarose gel to assess the integrity of the purified RNA. The RNA yield was 8.2 ± 2.7 μg/10^6^ cells (mean ± SD).

Before Reverse Transcription, residual genomic DNA was removed from Total RNA using DNaseI (Thermo Scientific). The reaction mix was as follows: 1 μg of Total RNA, 1 μl of 10x Reaction Buffer, 1 U of DNaseI and water up to 10 μl. The mix was incubated at 37°C for 30 min and stopped by adding 1 μl of EDTA 50 mM and incubating at 65°C for 10 min. A High Capacity cDNA Reverse Transcription kit (Applied Biosystem) was used for reverse transcription of mRNAs. The reaction mix was prepared as follows: 2 μg of total RNA, 2 μl of 10x RT Buffer, 0.8 μl of dNTP, 2 μl of 10x Random Hexamer, 50 U of MultiScribe Reverse Transcriptase, 1 μl of RNase Inhibitor and water up to 20 μl. cDNA synthesis was performed in a thermal block cycler (Bio-rad) following these steps: priming at 25°C for 10 min, transcription at 37°C for 120 min and enzyme inactivation at 85°C for 5 min. All cDNA samples were stored at -20°C pending qPCR analysis.

A qPCR assay was carried out in an Eppendorf Mastercycler EP Realplex (Eppendorf AG). Briefly, preliminary PCR reactions were run to optimize the concentration and ratio of each primer set. For all the cDNA templates 2 μL was used in a 20 μL qPCR amplification system of the SYBR Green Real Master Mix Kit. Primers for human WIP-1 gene and GAPDH as reference gene were designed using GeneWorks software (IntelliGenetix, Inc., Mountain View, CA, United States).

The primer pairs used were as follows: WIP-1 (NM003620) forward-5′-TTTTTATATTGTTTTTAGGTTATT-3′ and reverse-5′-ATCTATATAAACTTTTAACTCAATC-3′; GAPDH (NM002046) forward-5′-ACCACCATGGAGAAGGC-3′ and reverse-5′-GGCATGGACTGTGGTCATGA-3′. The amplification procedures and data computation followed were similar to those described above (Patruno et al., 2012). In this study stability of GAPDH did not vary in the cells whatever the conditions. Thus, the relative expression of WIP-1 was normalized to GAPDH using the ΔC_q_ method [relative expression = 2^-ΔCq^, where ΔC_q_ = C_q(WIP1)_ – C_q(GAPDH)_].

### Western Blotting

Peripheral Blood Mononuclear Cells were washed once in cold phosphate-buffered saline (PBS; 0.5 mol/L sodium phosphate, pH 7.5), harvested by gentle scraping, and used to prepare total or nuclear protein extracts. Total protein extracts were prepared as described previously by treating cells with lysis buffer for 30 min at 4°C (Felaco et al., 2000). Nuclear extracts were prepared as previously described (Speranza et al., 2009). The protein concentrations of the extracts were determined using the Bradford method (Bio-Rad protein assay, Hercules, CA, United States).

For Western blotting (WB) analysis, 50 μg of protein per lane was separated on a 4-12% NuPAGE gradient gel (Gibco Invitrogen), electrotransferred onto a nitrocellulose membrane and blocked with 10% skimmed milk in PBS containing 0.1% Tween-20 (Franceschelli et al., 2011). Blots were probed and incubated overnight at 4°C with polyclonal rabbit IgG anti-WIP1, and pNF-κB (p-p65^Ser536^) (Santa Cruz Biotechnology, Santa Cruz, CA, United States), all at 0.2 μg mL^-1^ in Tris-buffered saline (TBS)/0.1% Tween-20. A mouse antihuman monoclonal antibody recognizing human β-actin (A5441; Sigma–Aldrich) was used as the control in all experiments. A rabbit antihuman antibody for β-tubulin was used as the control in experiments with nuclear extract proteins (Santa Cruz Biotechnology, Santa Cruz, CA, United States). Three microgram of human recombinant the WIP-1 protein was used as positive control (Lifespan Biosciences, Seattle, WA, United States). Fifty microgram of nuclear extract proteins from PBMCs previously treated with the LPS (10 μg/mL) and INFγ (20 ng/mL) was used as positive control for the detection of p-p65^Ser536^ (Pesce et al., 2014).

Blots were then washed and incubated for 1 h with goat antirabbit-horseradish peroxidase (Pierce Biotechnology, Rockford, IL, United States) diluted 1:10000 in TBS/0.1% Tween-20. Immunoblot signals were developed using Super Signal Ultra chemiluminescence detection reagents (Pierce Biotechnology). The blot images were analyzed by a gel analysis software package (Gel Doc 1000; Bio-Rad, Milan, Italy). Data are expressed as the mean ± SD intensity of optical density.

### Role of WIP-1 in Regulating NF-kB Signaling

In order to detect the role of the WIP-1 in regulating NF-κB signaling, the PBMCs were isolated and cultured during the study, as above described. The cultured cells were pre-treated or not treated with the WIP-1 inhibitor GSK2830371 for 1 h, and later the cells were stimulated with the LPS and INFγ. The LPS was used at 10 μg/mL, IFNγ at 20 ng/mL, and GSK2830371 at 10 μM (Sigma–Aldrich, St Louis, MO, United States) (Pesce et al., 2014).

### Plasma Sampling and Measurement of C, CRP, IL-1β, IL-18, and IL-6 Levels, and *In Vitro* Production of Cytokines

Plasma was obtained with blood centrifugation at 1500 × *g* for 7 min and kept frozen at -20°C from all participants. The amount of plasma circulating IL-1β, IL-18, IL-6, C, and the CRP was assayed with the use of specific ELISA development systems (Pierce, Rockford, IL, United States) in all individuals. The amount of IL-1β, IL-18, IL-6 from the culture supernatants of the PBMCs was assayed with the use of specific ELISA development systems (Pierce, Rockford, IL, United States) in older adults after 6 months of being on a MT program. The experiments were performed according to the manufacturer’s instructions.

Plates were scanned using a specialized Charge Coupled Device cooled tool. The integrated density values of the spots of known standards were used to generate a standard curve. Density values for unknown samples were determined by using the standard curve for each subject in order to calculate the real values in pg/mL. All steps were performed in duplicate and at room temperature. The IL-1β assay sensitivity was ≤1.0 pg/ml, for IL-18 it was ≤12.5 pg/mL, for IL-6 it was ≤1 pg/ml, for C it was ≤56.7 pg/mL, and for CRP ≤ 10.0 pg/ml. The values below the assay sensitivity have been excluded. The intra- and inter-assay reproducibility was >90%. Duplicate values that differed from the mean by more than 10% were considered suspect and were repeated.

### Statistical Analysis

The normal distributions of data were tested by the Skewness and Kurtosis measurement. All the variables considered, resulted distributed in a normal manner. The results were reported separately for each group: young adults, total older adults, older adults Control Group (CG) and older adults Experimental Group (EG). The Unpaired Student *t*-test was used to assess significant differences between the young group and total older adults. The unpaired Student *t*-test and Chi-squared test were applied to evaluate significant differences between the CG and the EG. The same statistical test was applied to evaluate the differences for biological variables. Pearson’s (r) co-efficient correlation was applied to assess the strength of the relationship between the basal levels of age, BMI, WIP-1, C, IL-1β, IL-18, IL-6, CRP and of the MMSE scores in older adult subjects. In order to assess the multi-collinearity of the variables, the diagnostic factors, Tolerance and Variance Inflation Factor (VIF) were considered. Accordingly, the MMSE scores were used as an dependent variable in a multiple regression analysis, whereas Age, as well as C, WIP-1, IL-1β, IL-18, and IL-6 were used as independent ones. The differences pre- and post-cognitive training were analyzed by the Paired samples *t*-test. Statistical analyses were performed using the SPSS 19.0 statistic (SPSS Inc., Chicago, IL, United States) for Windows (IBM). Results are described as means ± SD for each assessment performed in triplicate. All statistical tests were two-tailed and were evaluated at an alpha level of 0.05.

## Results

### The Relationship between Global Cognitive Functioning and Biological Variables in Older Adults

**Table 3** shows the bivariate correlations at T0 between Age, BMI, the plasmatic level of C, CRP, the pro-inflammatory cytokines IL-1β, IL-18, IL-6, the mRNA expression of the WIP-1 from *ex vivo* PBMCs, and MMSE score of all older adults (*n* = 62).

**Table 3:** Pearson (r) correlation between Age, Body Mass Index (BMI), Mini Mental State Examination (MMSE) score, Cortisol (C) and immune markers at baseline in apparently healthy older adults (*n* = 62).

	BMI	MMSE	C	WIP1	IL-1β	IL-18	IL-6	CRP
Age	–0.235	–0.303^∗^	0.345^#^	–0.368^#^	0.320^∗^	0.371^#^	0.327^∗^	0.013
BMI	_	0.032	0.043	0.147	0.190	0.034	0.254^∗^	–0.136
MMSE		_	–0.340^#^	0.322^#^	–0.298^∗^	–0.324^#^	–0.296^∗^	–0.024
C			_	–0.299^∗^	0.272^∗^	0.363^#^	0.252^∗^	0.161
WIP-1				_	–0.426^#^	–0.660^#^	–0.505^#^	0.158
IL-1β					_	0.463^#^	0.426^#^	–0.188
IL-18						_	0.340^#^	–0.189
IL-6							_	–0.206

*WIP-1, Wild type p53-induced phosphatase 1; IL, Interleukin; CRP, C-reactive protein. ^∗^*p* < 0.05; ^#^*p* < 0.01.*

The Age parameter correlated significantly as well as negatively with the WIP-1 mRNA expression (*r* = -0.368, *p* < 0.01), whereas it correlated positively with the IL-1β (*r* = 0.320, *p* < 0.05), the IL-18 (*r* = 0.371, *p* < 0.01), and the IL-6 (*r* = 0.327, *p* < 0.05). The BMI did not correlate with any variable considered, except for the IL-6 (*r* = 0.254, *p* < 0.05).

The MMSE score correlated positively to the WIP-1 (*r* = 0.322; *p* < 0.01), and negatively to C (*r* = 0.340; *p* < 0.01) and to all the pro-inflammatory cytokines analyzed. Once again, the plasmatic level of C at baseline was significantly as well as negatively associated to the WIP-1 mRNA expression, and positively associated to IL-1β, IL-18, and IL-6. The cytokines were strongly and positively associated with each other. None of the variables considered, correlated significantly with the CRP.

The correlation values obtained at T0 were not confirmed at T1 for Age and the MMSE score, Age and C, and Age as well as immune markers evaluated. The same significant loss also occurred when the correlation were analyzed for the EG at T1.

**Table 4** presents the multiple linear regression analysis results. The MMSE score was considered as a dependent variable. The variables which show significant correlation vs. MMSE score were considered as potential independent variables for the analysis. In order to assess multi-collinearity, the diagnostic factors, Tolerance and VIF were evaluated. As a consequence, none of the variables were excluded. The analysis was carried out using Age, C, WIP-1, IL-1β, IL-18, and IL-6 as independent variables that were added stepwise. The model including Age, C, and amount of the WIP-1 mRNA in *ex vivo* PBMCs resulted as the most significant, accounting for 13.4% of the MMSE variance [*F*_(1,60)_= 4.105; *p* = 0.010] (Supplementary Table 1).

**Table 4:** Regression analysis summary for biological variables predicting cognitive function.

Dependent variable	Independent variables	*R*^2^	*R*^2^ adjusted	*F*	ANOVA (*p*)
MMSE score	Age	0.085	0.069	5.478	0.023
MMSE score	Age, C	0.147	0.117	4.986	0.010
MMSE score	Age, C, WIP1	0.178	0.134	4.105	0.010
MMSE score	Age, C, WIP1, IL-1β	0.193	0.135	3.342	0.016
MMSE score	Age, C, WIP1, IL-1β, IL-18	0.194	0.121	2.655	0.032
MMSE score	Age, C, WIP1, IL-1β, IL-18, IL-6	0.207	0.119	2.347	0.044

*The MMSE score was regressed on Age, Cortisol (C), Wild type p53-induced phosphatase 1 (WIP-1), IL-1β, IL-18, IL-6 in an stepwise regression analysis (*n* = 62). All variables were considered at baseline. IL, Interleukin.*

### Neuropsychological Test

Cognitive functioning was directly assessed using a neuropsychological test battery. In 62 apparent older adults, the global cognitive function was measured by the MMSE at T0, and after 6 months of the MT program (T1). At the same time, verbal memory was assessed to measure the ability to retain (Short Term Memory, STM) and recall (Long Term Memory, LTM) verbal information using the RAVL Test. Visual memory was evaluated through the ROCF Test. Attention was assessed by the TMT A, the TMT B and the test of Attentive Matrices.

The results reported in **Table 4** show that the MMSE score significantly increased at T1 in EG when compared to their baseline score (28.3 ± 1.6 vs. 26.1 ± 1.7, *p* < 0.01). We also observed that the MMSE score of EG at T1 was significantly higher than those of CG (28.3 ± 1.6 vs. 26.4 ± 1.9, *p* < 0.01).

With close attention, we observed significant differences pre- and post- the MT program for attentive test targets. The medium number of targets attempted was augmented in the EG over time (48.8 ± 6.2 vs. 55.7 ± 5.1, *p* < 0.05), and between the EG and the CG at T1 (55.7 ± 5.1 vs. 50.2 ± 5.0, *p* < 0.05). The two groups did not differ significantly in their TMT A (*p* = 0.611) or TMT B (*p* = 0.684) performance concerning completion time at T0. However, the completion time was different between the two groups at the T1, in which it was lower in the EG both for the TMT A (35.1 ± 8.1 vs. 42.2 ± 11.5, *p* > 0.05), that the TMT B (66.2 ± 20.7 vs. 78.9 ± 27.7, *p* > 0.05), but it did not reach the significance. The EG did not show significant variation of scores when compared to themselves at T0 for the TMT A (35.1 ± 8.1 vs. 41.8 ± 10.5, *p* > 0.05), and B (66.2 ± 20.7 vs. 79.3 ± 28.4, *p* > 0.05). We again found that the RAVLT score followed the same trend over time observed in the EG for global cognitive assessment. In detail, the STM score significantly increased from 2.5 ± 1.2 to 3.4 ± 1.0 (*p* < 0.01), and the LTM significantly improved from 2.3 ± 1.2 to 3.3 ± 1.2 (*p* < 0.01). We did not observe any variation over time in the CG. Nonetheless, the EG showed a significantly higher RAVLT score for the STM at T1 when compared to CG, whereas the groups did not differ significantly for STM and for any neuropsychological measure at the baseline (**Table 5**).

**Table 5:** Neuropsychological assessment of global cognitive function, attention, verbal and visual memory from a Control Group (CG) and from an Experimental Group (EG) at baseline (T0) and also after 6 months of a Memory Training Program (T1).

Neuropsychological tests	CG-T0 (*n* = 28) (mean ± SD)	EG-T0 (*n* = 34) (mean ± SD)	CG-T1 (*n* = 28) (mean ± SD)	EG-T1 (*n* = 34) (mean ± SD)
Dementia (MMSE)	26.7 ± 1.7	26.1 ± 1.7	26.4 ± 1.9	28.3 ± 1.6^a,b^
**TMT**				
Part A (s)	41.3 ± 12.5	41.8 ± 10.5	42.2 ± 11.5	35.1 ± 8.1
Part B (s)	78.1 ± 29.0	79.3 ± 28.4	78.9 ± 27.7	66.2 ± 20.7
AM (targets)	48.2 ± 5.4	48.8 ± 6.2	50.2 ± 5.0	55.7 ± 5.1^a,b^
**RAVLT**				
STM	2.6 ± 1.5	2.5 ± 1.2	2.7 ± 1.3	3.4 ± 1.0^a,b^
LTM	2.8 ± 1.1	2.3 ± 1.2	2.6 ± 1.3	3.3 ± 1.2^a,b^
**ROCF**				
Copy	26.8 ± 7.1	27.2 ± 8.2	27.5 ± 9.3	32.4 ± 5.8^a,b^
Delayed recall	12.4 ± 5.1	11.8 ± 5.0	13.2 ± 7.8	14.4 ± 3.8^a,b^

*The MMSE was used to quantify global cognitive functioning and cognitive change over time. The Attentive Matrices Test (AM) was used to measure selective and sustained attention (max targets = 60). The Trail Making Test (TMT) (Part A and Part B) provides information on visual search, the speed of processing, mental flexibility, selective visual attention and the ability of shifting. The score is represented by the time spent on test completion. The ability to retain (Short Term Memory, STM) and recall (Long Term Memory, LTM) verbal information was measured using the Rey Auditory Verbal Learning Test (RAVLT). The Rey-Osterrieth Complex Figure (ROCF) Test was used to assess visuo-spatial constructional functions, visuo-graphic memory, and a few planning aspects.*

*^a^*p* < 0.05 vs. CG at the same time (Unpaired Student *t*-test);*

*^b^*p* < 0.05 vs. themselves at T0 (Paired *t*-test).*

The data obtained for Visual memory through the ROCF Test, suggested that the MT program also significantly improved this function in subjects of the EG over time. We did not observe any difference between groups at the T1 (**Table 5**).

### Age Dependent WIP-1 mRNA and Protein Expression

**FIGURE 2:**
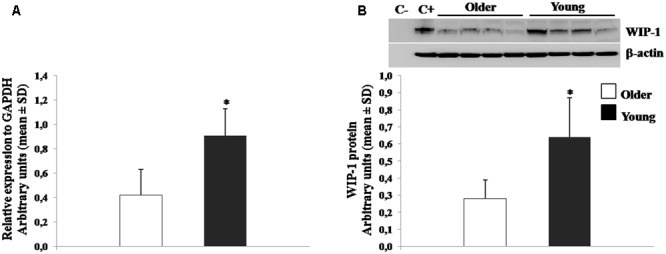
Wild type p53-induced phosphatase 1 (WIP-1) expression levels in freshly isolated Peripheral Blood Mononuclear Cells (PBMCs) from Young and Older adults at baseline. **(A)** mRNA was quantified using qPCR in Young adults (*n* = 31) and Older adults (*n* = 62). Sequence-specific primers to the WIP-1 gene were used to quantify the expression of WIP-1 promoter. Values are expressed as relative expression to GAPDH as the reference gene. Values are means ± SD of three experiments performed in triplicate. **(B)** WIP-1 protein expression in PBMCs from Young adults as compared to Older adults at T0. Top, representative Western blot image of WIP-1 and β-actin. Bottom, each immunoreactive band was analyzed by densitometry and normalized to β-actin levels. Each value is a mean ± SD of three different experiments performed in triplicate. ^∗^*p* < 0.05 vs. older adults.

The expression of WIP-1 has been shown to decrease during aging in murine model (Wong et al., 2009). In order to confirm on human the differentiated expression of the WIP-1 during age, qPCR, and WB experiments were performed to detect the mRNA and protein level, respectively, on freshly isolated PBMCs from healthy young and older adults. The results showed in **Figure 2**, suggest that older adults were characterized by significantly lower levels of the WIP-1 expression.

### Memory Training Modulation on WIP-1 mRNA and Protein Expression

**FIGURE 3:**
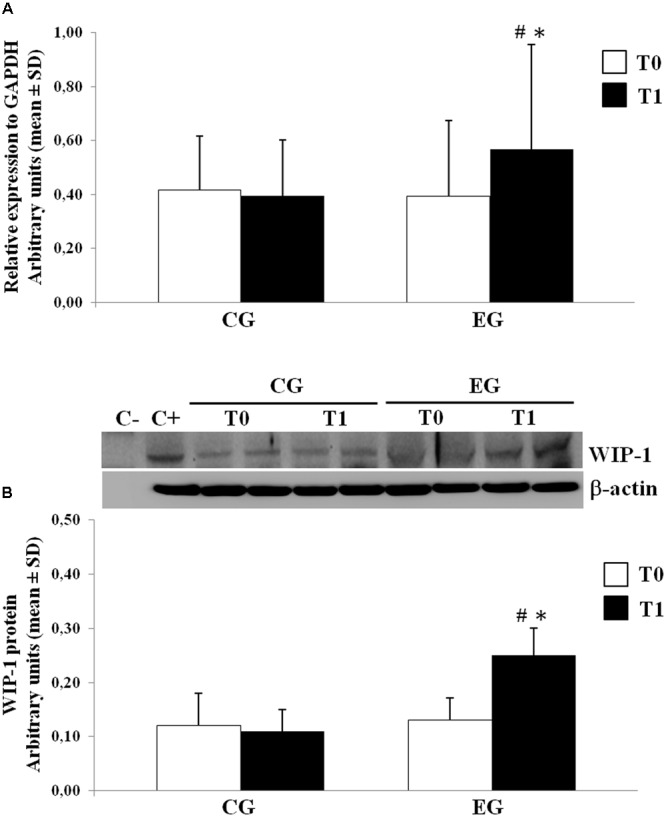
Wild type p53-induced phosphatase 1 expression levels in freshly isolated PBMCs from Control Group (CG) and Experimental Group (EG) at baseline and after 6 months of memory training program. **(A)** mRNA was quantified using qPCR in CG (*n* = 28) and EG (*n* = 34). Sequence-specific primers to the WIP-1 gene were used to quantify the expression of WIP-1 promoter. Values are expressed as relative expression to GAPDH as the reference gene. **(B)** WIP-1 protein expression in PBMCs from EG at T1 as compared to CG and EG at baseline (T0). Top, representative Western blot image of WIP-1 and β-actin. Bottom, each immunoreactive band was analyzed by densitometry and normalized to β-actin levels. Each value is a mean ± SD of three different experiments performed in triplicate. ^∗^ *p* < 0.05 vs. T0; ^#^ *p* < 0.05 vs. CG at the same time.

We determined the expression of the WIP-1 mRNA transcripts in freshly isolated PBMCs from selected healthy older adults by the qPCR at the baseline and after 6 months of the MT program in the CG and in the EG. **Figure 3A** shows the results from subjects of both groups. Compared with the CG, the PBMCs obtained from the EG were found to contain much higher levels of the mRNA for the WIP-1 at T1, determined as its expression relative to the reference gene GAPDH obtained from all subjects at each time. We did not observe any difference between groups at T0. Nevertheless, the WIP-1 mRNA expression was significantly higher in the PBMCs of the EG at T1 when compared to their value at the baseline. In detail, the detected amount of the WIP-1 mRNA in PBMCs from subjects of the EG at T1, is increased by more than 40% when compared to the EG at T0, and the CG at T1.

The levels of the WIP-1 protein in PBMCs were measured by the WB. In accordance with the mRNA levels, the PBMCs from the CG had significantly lower levels of the WIP-1 protein versus the EG after the MT program. The expression of the WIP-1 protein was also significantly increased within the EG over time. According to those reported for the mRNA, the protein level did not significantly differ between groups at T0 (**Figure 3B**).

### Cortisol, CRP, and Cytokines Production

In order to highlight the effects of the MT on the C level and the low-grade of inflammation which characterizes healthy older adults, the ELISA test was used to detect the plasmatic level of C as well as determining the non-specific marker for inflammation CRP in both groups at baseline and after the MT. Significant variation for CRP was not observed. As reported in **Table 6**, our data suggest that the MT significantly modulates the C plasma level. That level had significantly decreased in the EG at T1 when compared vs. CG at T1 and vs. EG at T0. In order to investigate any involvement of the MT on cytokine production in healthy older adults, the plasma levels of the IL-1β, the IL-18, and the IL-6 were evaluated. **Table 5** also shows the concentrations of cytokines detected in plasma of both groups at T0 and T1. The levels of the IL-1β, the IL-18 and the IL-6 detected at T0 did not significantly differ between the CG and the EG. On the other hand, the submission to the MT program significantly decreased the plasmatic level of all cytokines analyzed at T1 in the EG. Consequently, the subjects of this group showed a significant decrease over time.

**Table 6:** Time-dependent effect of memory training on Cortisol and the plasmatic pro-inflammatory mediators.

	CG-T0 (mean ± SD)	EG-T0 (mean ± SD)	CG-T1 (mean ± SD)	EG-T1 (mean ± SD)
Cortisol (pg/mL)	136.2 ± 56.4	134.9 ± 58.2	131.5 ± 67.7	109.3 ± 48.1^a,b^
IL-1β (pg/mL)	26.1 ± 8.3	25.9 ± 8.7	25.9 ± 7.9	22.8 ± 8.4^a,b^
IL-18 (pg/mL)	239.2 ± 48.0	241.8 ± 45.1	238.0 ± 41.0	212.1 ± 43.1^a,b^
IL-6 (pg/mL)	1.41 ± 0.26	1.46 ± 0.26	1.40 ± 0.27	1.23 ± 0.16^a,b^

*Cortisol, IL-1β, IL-18, and IL-6 plasma level are expressed as means ± SD (IL-1β, IL-18, and IL-6 CG at T0 and T1, *n* = 28; IL-1β, IL-18, and IL-6 EG at T0, *n* = 34; IL-1β and IL-18 EG at T1, *n* = 34; IL-6 EG, *n* = 31). T0, baseline time; T1, 6 months from the baseline time; CG, Control Group; EG, Experimental Group; IL, Interleukin.*

*^a^*p* < 0.05 vs. T0; ^b^*p* < 0.05 vs. CG at the same time.*

### The Role of WIP-1 in Regulating NF-kB Signaling in PBMCs from Healthy Older Adults

**FIGURE 4:**
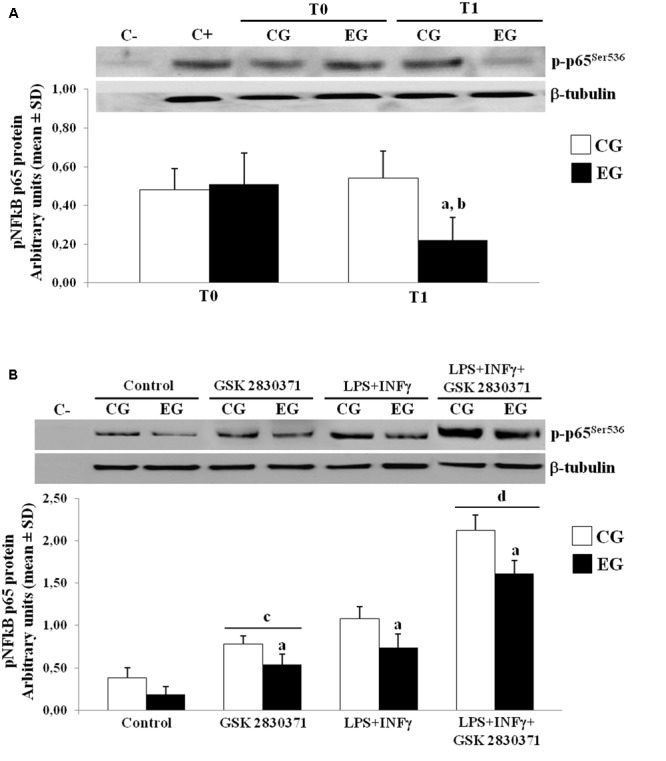
Phosphorylated levels of p65 in nuclei of PBMCs of older adults. **(A)** p-p65^Ser536^ protein expression in nuclei of freshly isolated PBMCs from CG and EG at T1 as compared to baseline values (T0). Top, representative Western blot image of p-p65^Ser536^. **(B)** PBMCs from CG and EG were cultured and then treated with WIP-1 inhibitor (GSK 2830371, 10 μM), LPS (10 μg/ml) + INFγ (20 ng/ml) or WIP-1 inhibitor and LPS (10 μg/ml) + INFγ (20 ng/ml), as described in “Materials and Methods” Section. Western immunoblotting was performed on nuclear proteins with antibody anti-p-p65^Ser536^ and anti-β-tubulin as the control. The image represents six different gels performed twice. Values are expressed relative to control (mean ± SD). ^a^*p* < 0.05 vs. CG; ^b^*p* < 0.05 vs. EG at T0; ^c^*p* < 0.05 vs. control PBMCs; ^d^*p* < 0.05 vs. PBMCs treated with LPS and INFγ.

The role of the NF-κB in the expression of pro-inflammatory cytokines has been broadly reviewed (Chung et al., 2011). The post-translational modification of the NF-κB (p65) affects its transcriptional activity; in particular, several reports have documented negative regulation of the NF-κB by the WIP-1, also through the direct dephosphorylation of Ser-536 of p65 (Chew et al., 2009). We next further performed WB studies to determine the phosphorylation of p65 at Ser-536, using specific phospho-p65 antibody on nuclear protein extracts. In the EG, we observed a down-regulation of nuclear translocation of the p-p65^Ser536^ as compared to the CG group at T1. As a result, the phosphorylated p65 in the EG showed low levels at T1 when compared to their own levels at T0 (**Figure 4A**).

In order to better investigate the role of this phosphatase in regulating the NF-κB nuclear translocation, the PBMCs from the CG and the EG at T0 and T1 were cultured and treated with a selective inhibitor of the WIP-1 (GSK 2830371) and/or LPS + INFγ, while nuclear protein extracts were detected by an antibody specific for the p-p65. As shown in **Figure 4B**, treatment with the LPS + INFγ increased the p-p65^Ser536^ expression in nuclei. This response was significantly elevated in the PBMCs from the CG as compared to the EG at T1. It should also be noted that the WIP-1 inhibition resulted in a significant increase in the constitutive and the LPS + INFγ-induced p65 phosphorylation and nuclear translocation in both groups. These findings suggested that the WIP-1 expression is significantly involved in the negative regulation of the NF-κB activity.

Furthermore, the inhibition of the activity of the WIP-1 resulted in a significantly higher constitutive and induced the release of the IL-1β, of the IL-18 and of the IL-6 in the medium of cultured cells (**Table 7**). We did not observe any significant differences between cells treated with an inhibitor and a medium alone, either in the p-p65^Ser536^ nuclear levels (**Figure 4B**) or in cytokines release (**Table 7**).

**Table 7:** Comparison of the IL-1β, of the IL-18, and of the IL-6 release in conditioned medium by non-stimulated and the LPS + INFγ and/or WIP-1 inhibitor (GSK2830371, 10 μM) treated Peripheral Blood Mononuclear Cells (PBMCs) (pg/ml/10^6^ cells) from 28 subjects of Control Group (CG) and 34 of the Experimental Group (EG) after 6 months of being on a Memory Training Program (T1).

	Control (pg/mL, mean ± SD)		WIP-1 inhibitor (pg/mL, mean ± SD)		LPS/INFγ (pg/mL, mean ± SD)		LPS/INFγ + WIP-1 inhibitor (pg/mL, mean ± SD)	
	CG	EG	CG	EG	CG	EG	CG	EG
IL1β	9.7 ± 4.2	5.6 ± 3.1^a^	35.9 ± 4.4	22.4 ± 6.2^a,b^	398.2 ± 37.4	321.4 ± 25.4^a^	512.0 ± 58.7	380.6 ± 54.8^a,b^
IL-18	36.9 ± 5.4	26.2 ± 6.1^a^	76.1 ± 6.8	51.7 ± 14.4^a,b^	282.6 ± 18.7	212.6 ± 16.1^a^	371.2 ± 22.2	324.1 ± 31.5^a,b^
IL-6	128.7 ± 25.4	94.1 ± 18.9^a^	156.2 ± 26.6	135.8 ± 19.2^a,b^	213.8 ± 22.7	178 ± 28.1^b^	244.6 ± 32.3	212.8 ± 0.3^a,b^

*Supernatants were collected, stored at -80°C until analysis, and then measured for IL-1β, IL-18, and IL-6 concentration using a commercial enzyme-linked immunosorbent assay (ELISA). All samples for a given assay were analyzed in duplicate at the same time. The ELISA values have an error range lower than 10%. The variation coefficient both inter-assay and intra-assay was <5%.*

*Values are presented as means ± SD. ^a^*p* < 0.05 vs. respective CG; ^b^*p* < 0.05 vs. respective WIP-1 inhibitor no treated cells.*

Altogether, these findings suggest a role for the WIP-1 activity in mediating the NF-κB nuclear levels and pro-inflammatory cytokines released in older adults. Furthermore, the submission to 6 months of the MT significantly restored the WIP-1 inhibitory function.

## Discussion

In the current study, we assume that the MT through mnemonic strategies could be considered as treatment that similarly to other stimuli of the EE, could enhance the cognitive and physical well-being of older adults. We studied a sample of apparently healthy older adults and used a longitudinal approach to test the hypothesis that the MT program could counteract the higher level of plasmatic C and sterile inflammation that characterizes adulthood as well as a decline in the cognitive function. We also investigated whether the phosphatase WIP-1 in *ex vivo* PBMCs could be considered a “master” molecule involved in modulating the level of plasmatic pro-inflammatory cytokines IL-6, IL-1β, and IL-18.

Concerning our correlation’s data, the associations recorded between cytokines, Age, MMSE score and mRNA of WIP-1 suggested a role of this phosphatase in the inhibition of the release of the IL-6, the IL-1β, and the IL-18. We first purposed a significant connection between global cognitive functioning and the IL-18 in healthy subjects, and highlighted a previously unrecognized relationship between plasmatic C levels and immune markers analyzed. Our results again reinforced the positive correlation between Age and C, as well as the negative association between the global cognitive function and C (Carvalhaes-Neto et al., 2003; Otte et al., 2005). Worth noting is that in a linear regression analysis, Age, C, and the WIP-1 mRNA expression resulted as the best predictors of variance in global cognitive performance and explained approximately the 13% variance in the MMSE score. We were in accordance with previous findings suggesting positive association between the cytokines measured and Age (Dinarello, 2006; Miles et al., 2008), and the IL-6 and BMI (Mohamed-Ali et al., 1997). We also agree with other authors in observing any significant association between the CRP plasmatic level and global cognitive functioning (Elderkin-Thompson et al., 2012).

As far as MT is concerned, accordingly with other studies, our results highlight better performance relating to verbal and visuo-spatial memory after 6 months of laboratory participation. Interestingly, as suggested by a significant decrease in the completion time of attentive matrices, MT contributes to attention performance enhancement. In regards to this, we are in line with the assumption that working memory used effectively is necessary to continuously select what is being represented by paying major attention to the way in which the working memory functions, and to update what is represented according to the relevance of the task (Palladino et al., 2001).

Cognitive improvement was paralleled over time by the lowering of the plasmatic level of the glucocorticoid hormone C, and by a significant reduction in the plasmatic levels of the pro-inflammatory cytokines evaluated. These findings suggest a previously unrecognized beneficial effect of the MT program participation, as increased C levels have been associated to several age-related pathologies that include muscle atrophy (Dohi et al., 2005), osteoporosis/hypercalcemia (Hetyey et al., 2007), hyperglycemia/hyperlipidemia, atherosclerosis, type II diabetes and major depression (Bindu et al., 2005; Leggio et al., 2005; Bauer, 2008). The peripheral level of the IL-6 contributes to age-related neural atrophy (Willette et al., 2010), it is considered a marker of increased risk of frailty (Ershler and Keller, 2000), and is associated with reduced muscle strength (Barbieri et al., 2003) during adulthood. No less important, the circulating levels of the IL-1β and of the IL-18 which are considered detrimental for aging successfully were involved in the mediation of several downstream effects of the NLRP3 inflammasome activation (Martinon et al., 2009; Youm et al., 2013). In particular, the IL-1β is thought to play a predominant role in the development of several age-related degenerative diseases including type 2 diabetes and Alzheimer’s Disease (AD) (Youm et al., 2011; Heneka et al., 2013). Both cytokines have been suggested to regulate obesity-induced inflammation, insulin resistance, and plaque instability and atherogenesis (Stienstra et al., 2011; Vandanmagsar et al., 2011; Wen et al., 2011; Shi et al., 2015).

At the same time, the MT could contribute to generate a positive loop in which memory performance improvement was further sustained by the lowering of the plasmatic level of C and of pro-inflammatory cytokines. In this respect, the higher level of C was related to the parameters of cognitive frailty (Carvalhaes-Neto et al., 2003), and more recently has been directly associated to cognitive performance in a large sample of non-demented subjects aged at least 65 years (Ouanes et al., 2017). Nevertheless, the IL-6 has been associated with poorer performance on tests of encoding and the recall of information and working memory (Elderkin-Thompson et al., 2012). The concentrations of the IL-1β were extensively related to the negative effects on memory in animal and human studies (Yirmiya and Goshen, 2011). To the best of our knowledge, there are no studies that have been previously associated with the IL-18 to some domain of cognition in healthy subjects. However, Zhang et al. (2013) suggests a role of a higher plasmatic level of this cytokine in determining the Visuo-spatial/Constructional deficit that characterizes naïve schizophrenic patients and has resulted in its circulatory level being significantly and positively associated with cognitive impairment in AD patients (Bossù et al., 2008).

The higher plasma levels of the IL-6, the IL-1β, and the IL-18 contribute to explain the more elevated NF-κB activation observed in freshly isolated PBMCs at the baseline vs. the EG after the MT. Our data prompted the fact that the MT inhibition of pro-inflammatory cytokines release from the PBMCs could be influenced by the restoration of the WIP-1 expression.

Our observations on human are consistent with the previous finding on rodents, in which the gene expression of WIP-1 resulted lower during aging (Wong et al., 2009). The PBMCs from older adults at the baseline, in which the WIP-1 expression was lower, were characterized by the higher p65^Ser536^ nuclear translocation when compared to the PBMCs from older adults treated with the MT in which the WIP-1 expression and activity was enhanced.

The analyses of cultured *ex vivo* PBMCs have shown that the WIP-1 could suppress the release of targets of the transcription factor NF-κB (i.e., cytokines medium release), with or without the LPS/INFγ stimulation. Our data also suggests that cultured PBMCs from older adults after the MT participation, were less sensitive toward the LPS/INFγ stimulation.

We are in accordance with the previous study which suggested an inhibiting role of the WIP-1 to the NF-κB signaling pathway. As showed by Chew et al. (2009), the over-expression of the WIP-1 or silencing it reduced or increased the NF-κB activation downstream treatment with the IL-1β. Once again, it was purposed that the enhancement of the NF-κB activity by the WIP-1 knockdown was strictly due to the levels of phosphorylation of the p65 on the Ser536. We can conclude that the WIP-1 could reduce the release of these cytokines by the inhibition of the NF-κB through direct de-phosphorylation of the p65 sub-unit on the Ser536.

Summarizing, our data confirms the validity of the MT, through mnemonic strategies in counteracting the aging associated decline of verbal, visual memory and of attention. In addition, we first suggested that this method of cognitive training contributes also to improve the other features of the phenotype of older adults by the decreasing level of the circulating C and pro-inflammatory cytokines and inflammation. Nevertheless, we suggest that the effect exerted on cytokines by MT could be due to the restoration of a higher level of the WIP-1 expression and activity.

The main limitation of the study is regards “How” the MT can contribute in restoring the WIP-1 expression and decreasing the pro-inflammatory cytokines level. Considering the associations observed between C, MMSE score, cytokines, and WIP-1 expression, we hypothesized a mechanism in which the lowering of this hormone could represent a potential link between cognitive stimulation and the observed decrease of low-grade inflammation that characterizes older adults.

The MT is a practice based on working memory tasks, which involves the hippocampus and its connections (Chengyang et al., 2016). At the present, we speculate that the MT could contribute to restore the inhibiting role on the HPA axis activity (Herman et al., 2003). It is indeed known that the hippocampal type I receptors were strictly expressed in the hippocampus and resulted diminished in aging. These receptors mediated the feedback control of the HPA axis, through the activation of tonic inhibitory projections mediated by GABAergic neurons proceeded to the Para Ventricular Nucleus of the hypothalamus (Swanson, 1991; Herman et al., 1996). To follow, the effect of MT on the diminishing C medium level, could determine the modulations on pro-inflammatory cytokines secretion recorded by analysis of *ex vivo* PBMCs. To this end, recent findings suggested that *in vivo* administration of C could enhance the migratory response of human monocytes and decrease the mRNA level of the IkBα protein, leading to higher nuclear levels of the p65 sub-unit of NF-kB (Yeager et al., 2016). Other studies support the existence of a positive feedback between GCs secretion and the activation of the Toll Like Receptor pro-inflammatory signaling pathway that was mediated by the synergistic action of GCs and pro-inflammatory stimuli (Bornstein et al., 2004; Hermoso et al., 2004). GCs have also been shown to induce the expression of NLRP3 inflammasome, in both cultured and primary macrophages, leading to higher secretion of IL1β, and IL-6 (Busillo et al., 2011). These results provide a potential explanation for some of the negative effects of higher level of C in older adults (Cruz-Topete and Cidlowski, 2015).

With regards to the WIP-1 expression, further analysis as to whether its expression in *ex vivo* PBMCs could be modulated by *in vivo* administration of C in young and older adults with or without synergy with pro-inflammatory stimulation will be done in the future. Furthermore, it will be of interest to investigate the age-related promoter status methylation of the PPM1D gene to test the hypothesis that an epigenetic mechanism (e.g., hypermethylation of PPM1D promoter κB site) could contribute to the WIP-1 repression, and could explain the progressive long life reduction of the WIP-1 expression previously observed in several cell types by other studies (Wong et al., 2009; Zhu et al., 2009).

## Conclusion

Our work provides insight some new properties of the MT in counteracting aging and a purposely planned WIP-1 deficiency as a part of its pathophysiology, bearing in mind that this phosphatase exerts an important role within the mechanisms that generally act in order to counteract and reduce the negative effects of environmental agents and those which were impaired during adulthood.

## Author Contributions

Conceived and designed the experiments: MP, MF, and AG. Performed the experiments: RT, ILF, AR, AF, SF, CT, and GC. Analyzed the data: MP, RT, MCV, MDL and LS. Wrote the manuscript: MP and RT.

## Conflict of Interest Statement

The authors declare that the research was conducted in the absence of any commercial or financial relationships that could be construed as a potential conflict of interest. The reviewer VB and handling Editor declared their shared affiliation, and the handling Editor states that the process nevertheless met the standards of a fair and objective review.

## References

[B1] Ablij H. C. Meinders A. E. (2002). C-reactive protein: history and revival. *Eur. J. Intern. Med.* 13 412–422. 10.1016/S0953-6205(02)00132-2 12384129

[B2] Baltes P. B. Baltes M. M. (1990). “Psychological perspectives on successful aging: the model of selective optimization with compensation,” in *Successful Aging* eds Baltes P. B. Baltes M. M. (Cambridge: University Press) 1–34. 10.1017/CBO9780511665684

[B3] Barbieri M. Ferrucci L. Ragno E. Corsi A. Bandinelli S. Bonafè M. (2003). Chronic inflammation and the effect of IGF-I on muscle strength and power in older persons. *Am. J. Physiol. Endocrinol. Metab.* 284 E481–E487. 10.1152/ajpendo.00319.2002 12419777

[B4] Bauer M. E. (2008). Chronic stress and immunosenescence: a review. *Neuroimmunomodulation* 15 241–250. 10.1159/000156467 19047801

[B5] Baumans V. (2005). Environmental enrichment for laboratory rodents and rabbits: requirements of rodents, rabbits, and research. *Ilar. J.* 46 162–170. 10.1093/ilar.46.2.162 15775025

[B6] Biasucci L. M. (2004). CDC/AHA workshop on markers of inflammation and cardiovascular disease: application to clinical and public health practice. Clinical use of inflammatory markers in patients with cardiovascular diseases: a background paper. *Circulation* 110 560–567. 10.1161/01.CIR.0000148983.88334.80 15611382

[B7] Bindu B. Rekha J. Kutty B. M. (2005). Post insult enriched housing improves the 8-arm radial maze performance but not the Morris water maze task in ventral subicular lesioned rats. *Brain. Res.* 1063 121–131. 10.1016/j.brainres.2005.09.044 16324686

[B8] Bornstein S. R. Zacharowski P. Schumann R. R. Barthel A. Tran N. Papewalis C. (2004). Impaired adrenal stress response in Toll-like receptor 2-deficient mice. *Proc. Natl. Acad. Sci. U.S.A.* 101 16695–16700. 10.1073/pnas.0407550101 15546996 PMC534518

[B9] Bossù P. Ciaramella A. Salani F. Bizzoni F. Varsi E. Di Iulio F. (2008). Interleukin-18 produced by peripheral blood cells is increased in Alzheimer’s disease and correlates with cognitive impairment. *Brain Behav. Immun.* 22 487–492. 10.1016/j.bbi.2007.10.001 17988833

[B10] Bowers S. L. Bilbo S. D. Dhabhar F. S. Nelson R. J. (2008). Stressor-specific alterations in corticosterone and immune responses in mice. *Brain Behav. Immun.* 22 105–113. 10.1016/j.bbi.2007.07.012 17890050 PMC2175078

[B11] Busillo J. M. Azzam K. M. Cidlowski J. A. (2011). Glucocorticoids sensitize the innate immune system through regulation of the NLRP3 inflammasome. *J. Biol. Chem.* 286 38703–38713. 10.1074/jbc.M111.275370 21940629 PMC3207479

[B12] Carvalhaes-Neto N. Huayllas M. K. Ramos L. R. Cendoroglo M. S. Kater C. E. (2003). Cortisol, DHEAS and aging: resistance to cortisol suppression in frail institutionalized elderly. *J. Endocrinol. Invest.* 26 17–22. 10.1007/BF03345117 12602529

[B13] Caswell M. (1993). Effect of patient age on tests of the acute-phase response. *Arch. Pathol. Lab. Med.* 117 906–909. 7690224

[B14] Cavallini E. Pagnin A. Vecchi T. (2003). Aging and everyday memory: the beneficial effect of memory training. *Arch. Gerontol. Geriatr.* 37 241–257. 10.1016/S0167-4943(03)00063-3 14511850

[B15] Chengyang L. Daqing H. Jianlin Q. Haisheng C. Qingqing M. Jin W. (2016). Short-term memory deficits correlate with hippocampal-thalamic functional connectivity alterations following acute sleep restriction. *Brain Imaging Behav.* 10.1007/s11682-016-9570-1 [Epub ahead of print] 27444729

[B16] Chesnokova V. Pechnick R. N. Wawrowsky K. (2016). Chronic peripheral inflammation, hippocampal neurogenesis, and behavior. *Brain. Behav. Immun.* 58 1–8. 10.1016/j.bbi.2016.01.017 26802985 PMC4956598

[B17] Chew J. Biswas S. Shreeram S. Humaidi M. Wong E. T. Dhillion M. K. (2009). WIP1 phosphatase is a negative regulator of NF-kappaB signalling. *Nat. Cell. Biol.* 11 659–666. 10.1038/ncb1873 19377466

[B18] Chung S. Sundar I. K. Hwang J. W. Yull F. E. Blackwell T. S. Kinnula V. L. (2011). NF-κB inducing kinase, NIK mediates cigarette smoke/TNFα-induced histone acetylation and inflammation through differential activation of IKKs. *PLoS ONE* 6:e23488 10.1371/journal.pone.0023488 PMC3160853 21887257

[B19] Cruz-Topete D. Cidlowski J. A. (2015). One hormone, two actions: anti- and pro-inflammatory effects of glucocorticoids. *Neuroimmunomodulation* 22 20–32. 10.1159/000362724 25227506 PMC4243162

[B20] Curlik D. M. II Shors T. J. (2013). Training your brain: do mental and physical (MAP) training enhance cognition through the process of neurogenesis in the hippocampus? *Neuropharmacology* 64 506–514. 10.1016/j.neuropharm.2012.07.027 22898496 PMC3445739

[B21] De Beni R. (2009). *Psicologia dell’Invecchiamento.* Bologna: Il Mulino.

[B22] De Kloet E. R. Vreugdenhil E. Oitzl M. S. Joëls M. (1998). Brain corticosteroid receptor balance in health and disease. *Endocr. Rev.* 19 269–301. 10.1210/er.19.3.269 9626555

[B23] Deuschle M. Gotthardt U. Schweiger U. Weber B. Körner A. Schmider J. (1997). With aging in humans the activity of the hypothalamus-pituitary-adrenal system increases and its diurnal amplitude flattens. *Life Sci.* 61 2239–2246. 10.1016/S0024-3205(97)00926-0 9393943

[B24] Dinarello C. A. (2006). Interleukin 1 and interleukin 18 as mediators of inflammation and the aging process. *Am. J. Clin. Nutr.* 83 447S–455S. 16470011 10.1093/ajcn/83.2.447S

[B25] Ding Y. Gao Z. G. Jacobson K. A. Suffredini A. F. (2010). Dexamethasone enhances ATP-induced inflammatory responses in endothelial cells. *J. Pharmacol. Exp. Ther.* 335 693–702. 10.1124/jpet.110.171975 20826566 PMC2993554

[B26] Dohi K. Satoh K. Ohtaki H. Shioda S. Miyake Y. Shindo M. (2005). Elevated plasma levels of bilirubin in patients with neurotrauma reflect its pathophysiological role in free radical scavenging. *In Vivo* 19 855–860. 16097438

[B27] Elderkin-Thompson V. Irwin M. R. Hellemann G. Kumar A. (2012). Interleukin-6 and memory functions of encoding and recall in healthy and depressed elderly adults. *Am. J. Geriatr. Psychiatry* 20 753–763. 10.1097/JGP.0b013e31825d08d6 22892560 PMC3690956

[B28] Ershler W. B. Keller E. T. (2000). Age-associated increased interleukin-6 gene expression, late-life diseases, and frailty. *Annu. Rev. Med.* 51 245–270. 10.1146/annurev.med.51.1.245 10774463

[B29] Felaco M. Di Maio F. D. De Fazio P. D’Arcangelo C. De Lutiis M. A. Varvara G. (2000). Localization of the e-NOS enzyme in endothelial cells and odontoblasts of healthy human dental pulp. *Life Sci.* 68 297–306. 10.1016/S0024-3205(00)00935-8 11191645

[B30] Ferrari E. Magri F. (2008). Role of neuroendocrine pathways in cognitive decline during aging. *Ageing Res. Rev.* 7 225–233. 10.1016/j.arr.2008.07.001 18672097

[B31] Fiscella M. Zhang H. Fan S. Sakaguchi K. Shen S. Mercer W. E. (1997). Wip1, a novel human protein phosphatase that is induced in response to ionizing radiation in a p53-dependent manner. *Proc. Natl. Acad. Sci. U.S.A.* 94 6048–6053. 10.1073/pnas.94.12.6048 9177166 PMC20998

[B32] Folstein M. F. Folstein S. E. McHugh P. R. (1975). “Mini-mental state”. A practical method for grading the cognitive state of patients for the clinician. *J. Psychiatr. Res.* 12 189–198. 10.1016/0022-3956(75)90026-6 1202204

[B33] Franceschelli S. Pesce M. Vinciguerra I. Ferrone A. Riccioni G. Patruno A. (2011). Licocalchone-C extracted from Glycyrrhiza glabrainhibits lipopolysaccharide-interferon-γ inflammation by improving antioxidant conditions and regulating inducible nitric oxide synthase expression. *Molecules* 16 5720–5734. 10.3390/molecules16075720 21734629 PMC6264366

[B34] Franceschi C. (2007). Inflammaging as a major characteristic of old people: can it be prevented or cured? *Nutr. Rev.* 65(12 Pt 2) S173–S176. 10.1111/j.1753-4887.2007.tb00358.x 18240544

[B35] Franceschi C. Campisi J. (2014). Chronic inflammation (inflammaging) and its potential contribution to age-associated diseases. *J. Gerontol. A Biol. Sci. Med. Sci.* 69(Suppl. 1) S4–S9. 10.1093/gerona/glu057 24833586

[B36] Gabay C. Kashner I. (1999). Acute phase proteins and other systemic responses to inflammation. *N. Engl. J. Med.* 340 448–454. 10.1056/NEJM199902113400607 9971870

[B37] Geerlings M. I. Sigurdsson S. Eiriksdottir G. Garcia M. E. Harris T. B. Gudnason V. (2015). Salivary cortisol, brain volumes, and cognition in community-dwelling elderly without dementia. *Neurology* 85 976–983. 26291281 10.1212/WNL.0000000000001931 PMC4567466

[B38] Giordano R. Bo M. Pellegrino M. Vezzari M. Baldi M. Picu A. (2005). Hypothalamus-pituitary-adrenal hyperactivity in human aging is partially refractory to stimulation by mineralocorticoid receptor blockade. *J. Clin. Endocrinol. Metab.* 90 5656–5662. 10.1210/jc.2005-0105 16014406

[B39] Gorelick P. B. Scuteri A. Black S. E. Decarli C. Greenberg S. M. Iadecola C. (2014). American heart association stroke council, council on epidemiology and prevention, council on cardiovascular nursing, council on cardiovascular radiology and intervention, and council on cardiovascular Hannan AJ. Environmental enrichment and brain repair: harnessing the therapeutic effects of cognitive stimulation and physical activity to enhance experience-dependent plasticity. *Neuropathol. Appl. Neurobiol.* 40 13–25. 10.1111/nan.12102 24354721

[B40] Green D. R. Galluzzi L. Kroemer G. (2011). Mitochondria and the autophagy-inflammation-cell death axis in organismal aging. *Science* 333 1109–1112. 10.1126/science.1201940 21868666 PMC3405151

[B41] Hannan A. J. (2014). Environmental enrichment and brain repair: harnessing the therapeutic effects of cognitive stimulation and physical activity to enhance experience-dependent plasticity. *Neuropathol. Appl. Neurobiol.* 1 13–25. 10.1111/nan.12102 24354721

[B42] Heneka M. T. Kummer M. P. Stutz A. Delekate A. Schwartz S. Vieira-Saecker A. (2013). NLRP3 is activated in Alzheimer’s disease and contributes to pathology in APP/PS1 mice. *Nature* 493 674–678. 10.1038/nature11729 23254930 PMC3812809

[B43] Herman J. P. Figueiredo H. Mueller N. K. Ulrich-Lai Y. Ostrander M. M. Choi D. C. (2003). Central mechanisms of stress integration: hierarchical circuitry controlling hypothalamo-pituitary-adrenocortical responsiveness. *Front. Neuroendocrinol.* 24:151–180. 10.1016/j.yfrne.2003.07.001 14596810

[B44] Herman J. P. Prewitt C. M. Cullinan W. E. (1996). Neuronal circuit regulation of the hypothalamo-pituitary-adrenocortical stress axis. *Crit. Rev. Neurobiol.* 10 371–394. 10.1615/CritRevNeurobiol.v10.i3-4.50 8978987

[B45] Hermoso M. A. Matsuguchi T. Smoak K. Cidlowski J. A. (2004). Glucocorticoids and tumor necrosis factor alpha cooperatively regulate toll-like receptor 2 gene expression. *Mol. Cell. Biol.* 24 4743–4756. 10.1128/MCB.24.11.4743-4756.2004 15143169 PMC416411

[B46] Hetyey C. S. Manczur F. Dudás-Györki Z. Reiczigel J. Ribiczey P. Vajdovich P. (2007). Plasma antioxidant capacity in dogs with naturally occurring heart diseases. *J. Vet. Med. A Physiol. Pathol. Clin. Med.* 54 36–39. 10.1111/j.1439-0442.2007.00911.x 17359453

[B47] Jankowsky J. L. Melnikova T. Fadale D. J. Xu G. M. Slunt H. H. Gonzales V. (2005). Environmental enrichment mitigates cognitive deficits in a mouse model of Alzheimer’s disease. *J. Neurosci.* 25 5217–5224. 10.1523/JNEUROSCI.5080-04.2005 15917461 PMC4440804

[B48] Jin R. O. Mason S. Mellon S. H. Epel E. S. Reus V. I. Mahan L. (2016). Cortisol/DHEA ratio and hippocampal volume: a pilot study in major depression and healthy controls. *Psychoneuroendocrinology* 72 139–146. 10.1016/j.psyneuen.2016.06.017 27428086 PMC5203799

[B49] Kempermann G. Gast D. Gage F. H. (2002). Neuroplasticity in old age: sustained five fold induction of hippocampal neurogenesis by long-term environmental enrichment. *Ann. Neurol.* 52 135–143. 10.1002/ana.10262 12210782

[B50] Langlais D. Couture C. Balsalobre A. Drouin J. (2008). Regulatory network analyses reveal genome-wide potentiation of LIF signaling by glucocorticoids and define an innate cell defense response. *PLoS Genet.* 4:e1000224 10.1371/journal.pgen.1000224 PMC2562516 18927629

[B51] Lee B. K. Glass T. A. Wand G. S. McAtee M. J. Bandeen-Roche K. Bolla K. I. (2008). Apolipoprotein e genotype, cortisol, and cognitive function in community-dwelling older adults. *Am. J. Psychiatry* 165 1456–1464. 10.1176/appi.ajp.2008.07091532 18593777 PMC2579316

[B52] Leggio M. G. Mandolesi L. Frederico F. Spirito F. Ricci B. Gelfo F. (2005). Environmental enrichment promotes improved spatial abilities and enhanced dendritic growth in the rat. *Behav. Brain Res.* 163 78–90. 10.1016/j.bbr.2005.04.009 15913801

[B53] Lezak M. D. (1995). *Neuropsychological Assessment.* New York, NY: Oxford University Press.

[B54] Ligthart G. J. Corberand J. X. Fournier C. Galanaud P. Hijmans W. Kennes B. (1984). Admission criteria for immunogerontological stud- ies in man: the SENIEUR protocol. *Mech. Ageing Dev.* 28 47–55. 10.1016/0047-6374(84)90152-0 6513613

[B55] Lupien S. J. de Leon M. de Santi S. Convit A. Tarshish C. Nair N. P. (1998). Cortisol levels during human aging predict hippocampal atrophy and memory deficits. *Nat. Neurosci.* 1 69–73. 10195112 10.1038/271

[B56] Lupien S. J. Evans A. Lord C. Miles J. Pruessner M. Pike B. (2007). Hippocampal volume is as variable in young as in older adults: implications for the notion of hippocampal atrophy in humans. *Neuroimage* 34 479–485. 10.1016/j.neuroimage.2006.09.041 17123834

[B57] Maggio M. Basaria S. Ceda G. P. Ble A. Ling S. M. Bandinelli S. (2005). The relationship between testosterone and molecular markers of inflammation in older men. *J. Endocrinol. Invest.* 28(11 Suppl. Proceedings) 116–119. 16760639

[B58] Mangnus L. Nieuwenhuis W. P. van Steenbergen H. W. Huizinga T. W. Reijnierse M. van der Helm-van Mil A. H. (2016). Body mass index and extent of MRI-detected inflammation: opposite effects in rheumatoid arthritis versus other arthritides and asymptomatic persons. *Arthritis. Res. Ther.* 18 245 10.1186/s13075-016-1146-3 PMC5075146 27770823

[B59] Martinon F. Mayor A. Tschopp J. (2009). The inflammasomes: guardians of the body. *Annu. Rev. Immunol.* 27 229–265. 10.1146/annurev.immunol.021908.132715 19302040

[B60] Mendelson J. H. Goletiani N. Sholar M. B. Siegel A. J. Mello N. K. (2008). Effects of smoking successive low- and high-nicotine cigarettes on hypothalamic-pituitary-adrenal axis hormones and mood in men. *Neuropsychopharmacology* 33 749–760. 10.1038/sj.npp.1301455 17507912

[B61] Messner B. Bernhard D. (2014). Smoking and cardiovascular disease: mechanisms of endothelial dysfunction and early atherogenesis. *Arterioscler. Thromb. Vasc. Biol.* 34 509–515. 10.1161/ATVBAHA.113.300156 24554606

[B62] Metti A. L. Aizenstein H. Yaffe K. Boudreau R. M. Newman A. Launer L. (2015). Trajectories of peripheral interleukin-6, structure of the hippocampus, and cognitive impairment over 14 years in older adults. *Neurobiol. Aging* 36 3038–3044. 10.1016/j.neurobiolaging.2015.07.025 26279115 PMC4718400

[B63] Miles E. A. Rees D. Banerjee T. Cazzola R. Lewis S. Wood R. (2008). Age-related increases in circulating inflammatory markers in men are independent of BMI, blood pressure and blood lipid concentrations. *Atherosclerosis* 196 298–305. 10.1016/j.atherosclerosis.2006.11.002 17118371

[B64] Mohamed-Ali V. Goodrick S. Rawesh A. Katz D. R. Miles J. M. Yudkin J. S. (1997). Subcutaneous adipose tissue releases interleukin-6, but not tumor necrosis factor-alpha, in vivo. *J. Clin. Endocrinol. Metab.* 82 4196–4200. 9398739 10.1210/jcem.82.12.4450

[B65] Morris N. L. Ippolito J. A. Curtis B. J. Chen M. M. Friedman S. L. Hines I. N. (2015). Alcohol and inflammatory responses: summary of the 2013 Alcohol and Immunology Research Interest Group (AIRIG) meeting. *Alcohol* 49 1–6. 10.1016/j.alcohol.2014.07.018 25468277 PMC4314434

[B66] O’Hara R. Schröder C. M. Mahadevan R. Schatzberg A. F. Lindley S. Fox S. (2007). Serotonin transporter polymorphism, memory and hippocampal volume in the elderly: association and interaction with cortisol. *Mol. Psychiatry* 12 544–555. 10.1038/sj.mp.4001978 17353910 PMC2084475

[B67] Otte C. Hart S. Neylan T. C. Marmar C. R. Yaffe K. Mohr D. C. (2005). A meta-analysis of cortisol response to challenge in human aging: importance of gender. *Psychoneuroendocrinology* 30 80–91. 10.1016/j.psyneuen.2004.06.002 15358445

[B68] Ouanes S. Castelao E. Gebreab S. von Gunten A. Preisig M. Popp J. (2017). Life events, salivary cortisol, and cognitive performance in nondemented subjects: a population-based study. *Neurobiol. Aging* 51 1–8. 10.1016/j.neurobiolaging.2016.11.014 28012996

[B69] Palladino P. Cornoldi C. De Beni R. Pazzaglia F. (2001). Working memory and updating processes in reading comprehension. *Mem. Cognit.* 29 344–354. 10.3758/BF03194929 11352218

[B70] Patruno A. Franceschelli S. Pesce M. Maccallini C. Fantacuzzi M. Speranza L. (2012). Novel aminobenzyl-acetamidine derivative modulate the differential regulation of NOSs in LPS induced inflammatory response: role of PI3K/Akt pathway. *Biochim. Biophys. Acta* 1820 2095–2104. 10.1016/j.bbagen.2012.08.014 22951221

[B71] Pesce M. Ferrone A. Rizzuto A. Tatangelo R. Iezzi I. Ladu S. (2014). The SHP-1 expression is associated with cytokines and psychopathological status in unmedicated first episode schizophrenia patients. *Brain Behav. Immun.* 41 251–260. 10.1016/j.bbi.2014.04.008 24793756

[B72] Pesce M. Speranza L. Franceschelli S. Ialenti V. Iezzi I. Patruno A. (2013). Positive correlation between serum interleukin-1β and state anger in rugby athletes. *Aggress. Behav.* 39 141–148. 10.1002/ab.21457 23208827

[B73] Reedy S. D. Boone K. B. Cottingham M. E. Glaser D. F. Lu P. H. Victor T. L. (2013). Cross validation of the Lu and colleagues (2003) rey-osterrieth complex figure test effort equation in a large known-group sample. *Arch. Clin. Neuropsychol.* 28 30–37. 10.1093/arclin/acs106 23232864

[B74] Reitan R. M. (1958). Validity of the trail making test as an indicator of organic brain damage. *Percept. Mot. Skills* 8 271–276. 10.2466/pms.1958.8.3.271

[B75] Rosano C. Marsland A. L. Gianaros P. J. (2012). Maintaining brain health by monitoring inflammatory processes: a mechanism to promote successful aging. *Aging Dis.* 3 16–33. 22500269 PMC3320802

[B76] Salminen A. Kaarniranta K. (2011). Control of p53 and NF-κB signaling by WIP1 and MIF: role in cellular senescence and organismal aging. *Cell. Signal.* 23 747–752. 10.1016/j.cellsig.2010.10.012 20940041

[B77] Sher K. J. Bartholow B. D. Peuser K. Erickson D. J. Wood M. D. (2007). Stressresponse- dampening effects of alcohol: attention as a mediator and moderator. *J. Abnorm. Psychol.* 116 362–377. 10.1037/0021-843X.116.2.362 17516768 PMC2679524

[B78] Shi X. Sun H. Zhou D. Xi H. Shan L. (2015). Arctigenin attenuates lipopolysaccharide-induced acute lung injury in rats. *Inflammation* 38 623–631. 10.1007/s10753-014-9969-z 25008149

[B79] Simpson J. Kelly J. P. (2011). The impact of environmental enrichment in laboratory rats–behavioural and neurochemical aspects. *Behav. Brain Res.* 222 246–264. 10.1016/j.bbr.2011.04.002 21504762

[B80] Singhal G. Jaehne E. J. Corrigan F. Baune B. T. (2014). Cellular and molecular mechanisms of immunomodulation in the brain through environmental enrichment. *Front. Cell. Neurosci.* 8:97 10.3389/fncel.2014.00097 PMC3982075 24772064

[B81] Sox H. Liang M. (1986). The erythrocyte sedimentation rate. *Ann. Intern. Med.* 104 515–523. 10.7326/0003-4819-104-4-515 3954279

[B82] Speranza L. Franceschelli S. Pesce M. Menghini L. Patruno A. Vinciguerra I. (2009). Anti-inflammatory properties of the plant *Verbascum mallophorum*. *J. Biol. Regul. Homeost. Agents* 23 189–195. 19828096

[B83] Speranza L. Pesce M. Franceschelli S. Bucciarelli T. Gallina S. Riccioni G. (2013). The biological evaluation of ADMA/SDMA and eNOS in patients with ACHF. *Front. Biosci.* 5:551–557. 10.2741/e637 23277011

[B84] Spinnler H. Tognoni G. (1987). Italian group on the neuropsychological study of ageing: Italian standardization and classification of neuropsychological tests. *Ital. J. Neurol. Sci.* 6 1–120. 3330072

[B85] Stienstra R. van Diepen J. A. Tack C. J. Zaki M. H. van de Veerdonk F. L. Perera D. (2011). Inflammasome is a central player in the induction of obesity and insulin resistance. *Proc. Natl. Acad. Sci. U.S.A.* 108 15324–15329. 10.1073/pnas.1100255108 21876127 PMC3174591

[B86] Sudheimer K. D. O’Hara R. Spiegel D. Powers B. Kraemer H. C. Neri E. (2014). Cortisol, cytokines, and hippocampal volume interactions in the elderly. *Front. Aging Neurosci.* 6:153 10.3389/fnagi.2014.00153 PMC4079951 25071562

[B87] Sun B. Hu X. Liu G. Ma B. Xu Y. Yang T. (2014). Phosphatase Wip1 negatively regulates neutrophil migration and inflammation. *J. Immunol.* 192 1184–1195. 10.4049/jimmunol.1300656 24395919

[B88] Sutliffe J. T. Wilson L. D. de Heer H. D. Foster R. L. Carnot M. J. (2015). C-reactive protein response to a vegan lifestyle intervention. *Complement. Ther. Med.* 23 32–37. 10.1016/j.ctim.2014.11.001 25637150

[B89] Swanson L. W. (1991). Biochemical switching in hypothalamic circuits mediating responses to stress. *Prog. Brain Res.* 87 181–200. 10.1016/S0079-6123(08)63052-6 1866447

[B90] Tan X. Zhang J. Jin W. Li L. Xu W. Zheng H. (2013). Wip1 phosphatase involved in lipopolysaccharide-induced neuroinflammation. *J. Mol. Neurosci.* 51 959–966. 10.1007/s12031-013-0080-y 23959423

[B91] Tatomir A. Micu C. Crivii C. (2014). The impact of stress and glucocorticoids on memory. *Clujul. Med.* 87 3–6. 10.15386/cjm.2014.8872.871.at1cm2 26527987 PMC4462413

[B92] Travison T. G. O’Donnell A. B. Araujo A. B. Matsumoto A. M. McKinlay J. B. (2007). Cortisol levels and measures of body composition in middle-aged and older men. *Clin. Endocrinol. (Oxf)* 67 71–77. 10.1111/j.1365-2265.2007.02837.x 17466009

[B93] Vandanmagsar B. Youm Y. H. Ravussin A. Galgani J. E. Stadler K. Mynatt R. L. (2011). The NLRP3 inflammasome instigates obesity-induced inflammation and insulin resistance. *Nat. Med.* 17 179–188. 10.1038/nm.2279 21217695 PMC3076025

[B94] Vogel S. Fernández G. Joëls M. Schwabe L. (2016). Cognitive adaptation under stress: a case for the mineralocorticoid receptor. *Trends Cogn. Sci.* 20 192–203. 10.1016/j.tics.2015.12.003 26803208

[B95] Wen H. Gris D. Lei Y. Jha S. Zhang L. Huang M. T. (2011). Fatty acid-induced NLRP3-ASC inflammasome activation interferes with insulin signaling. *Nat. Immunol.* 12 408–415. 10.1038/ni.2022 21478880 PMC4090391

[B96] Willette A. A. Bendlin B. B. McLaren D. G. Canu E. Kastman E. K. Kosmatka K. J. (2010). Age-related changes in neural volume and microstructure associated with interleukin-6 are ameliorated by a calorie-restricted diet in old rhesus monkeys. *Neuroimage* 51 987–994. 10.1016/j.neuroimage.2010.03.015 20298794 PMC2877377

[B97] Wong E. S. Le Guezennec X. Demidov O. N. Marshall N. T. Wang S. T. Krishnamurthy J. (2009). p38MAPK controls expression of multiple cell cycle inhibitors and islet proliferation with advancing age. *Dev. Cell* 17 142–149. 10.1016/j.devcel.2009.05.009 19619499

[B98] Yeager M. P. Pioli P. A. Collins J. Barr F. Metzler S. Sites B. D. (2016). Glucocorticoids enhance the in vivo migratory response of human monocytes. *Brain Behav. Immun.* 54 86–94. 10.1016/j.bbi.2016.01.004 26790757 PMC4828285

[B99] Yesavage J. A. Brink T. L. Rose T. L. Lum O. Huang V. Adey M. (1982). Development and validation of a geriatric depression screening scale: a preliminary report. *J. Psychiatr. Res.* 17 37–49. 10.1016/0022-3956(82)90033-4 7183759

[B100] Yirmiya R. Goshen I. (2011). Immune modulation of learning, memory, neural plasticity and neurogenesis. *Brain Behav. Immun.* 25 181–213. 10.1016/j.bbi.2010.10.015 20970492

[B101] Youm Y. H. Adijiang A. Vandanmagsar B. Burk D. Ravussin A. Dixit V. D. (2011). Elimination of the NLRP3-ASC inflammasome protects against chronic obesity-induced pancreatic damage. *Endocrinology* 152 4039–4045. 10.1210/en.2011-1326 21862613 PMC3199005

[B102] Youm Y. H. Grant R. W. McCabe L. R. Albarado D. C. Nguyen K. Y. Ravussin A. (2013). Canonical Nlrp3 inflammasome links systemic low-grade inflammation to functional decline in aging. *Cell. Metab.* 18 519–532. 10.1016/j.cmet.2013.09.010 24093676 PMC4017327

[B103] Zhang X. Y. Tang W. Xiu M. H. De Yang F. Tan Y. L. Wang Z. R. (2013). Interleukin 18 and cognitive impairment in first episode and drug naïve schizophrenia versus healthy controls. *Brain Behav. Immun.* 32 105–111. 10.1016/j.bbi.2013.03.001 23499732

[B104] Zhu Y. H. Zhang C. W. Lu L. Demidov O. N. Sun L. Yang L. (2009). Wip1 regulates the generation of new neural cells in the adult olfactory bulb through p53-dependent cell cycle control. *Stem Cells* 27 1433–1442. 10.1002/stem.65 19489034

